# Alleviation of experimental arthritis in SKG mice through *Nr4a1* agonization

**DOI:** 10.3389/fimmu.2026.1758616

**Published:** 2026-02-25

**Authors:** Yoichi Nakayama, Ryosuke Hiwa, Ayaka Okubo, Mikihito Shoji, Mirei Shirakashi, Hideaki Tsuji, Koji Kitagori, Ran Nakashima, Shuji Akizuki, Hajime Yoshifuji, Akio Morinobu

**Affiliations:** 1 Department of Rheumatology and Clinical Immunology, Kyoto University Graduate School of Medicine, Kyoto, Japan; 2 Occupational Welfare Division, Agency for Health, Safety and Environment, Kyoto University, Kyoto, Japan

**Keywords:** Cytosporone B, experimental arthritis, NR4A, rheumatoid arthritis, SKG mice, T cell antigen receptor signaling, T cells

## Abstract

**Introduction:**

Rheumatoid arthritis (RA), a chronic autoimmune disease, is characterized by CD4^+^ T cell-mediated synovial inflammation, with T helper (Th)17 cells being implicated in RA pathogenesis. *Nr4a1* is an orphan nuclear receptor functioning as a negative regulator of T cell activation and central tolerance. Cytosporone B (CsnB) is a small-molecule agonist of *Nr4a1* and can exert immunomodulatory effects. However, its efficacy in T cell-driven autoimmune arthritis remains unclear. This study aimed to investigate the therapeutic effect of CsnB-mediated *Nr4a1* agonization on RA development in SKG mice and evaluate its impact on T cell function.

**Methods:**

The SKG mouse model of T cell-dependent chronic arthritis was constructed via zymosan A induction. The mice were intraperitoneally treated with CsnB, and disease severity and immune cell populations were evaluated by clinical scoring and flow cytometry. *In vitro* assays were performed to examine T cell antigen receptor (TCR)-induced T cell activation and Th17 differentiation. Additionally, RNA sequencing was performed to profile transcriptomic changes in CD4^+^ T cells following TCR stimulation.

**Results:**

CsnB markedly attenuated arthritis development and reduced the population of effector memory and Th17 cells in the spleen and synovium. Furthermore, *in vitro* assay results showed that CsnB suppressed T cell activation, downregulated interleukin (IL)-2 and activation markers, and repressed inflammatory gene expression. CsnB inhibited Th17 differentiation and IL-6–signal transducer and activator of transcription 3 signaling by reducing CD130 (*Il6st*) expression.

**Discussion:**

Altogether, the findings of this study showed that CsnB, one of the agonists of *Nr4a1*, suppressed TCR-driven T cell activation and Th17 differentiation, thereby ameliorating autoimmune arthritis in SKG mice. These findings highlight the potential of *Nr4a1* as an immunotherapeutic target in T cell-mediated autoimmune arthritis, particularly in RA subsets characterized by TCR signaling dysfunction.

## Graphical Abstract

**Figure:**
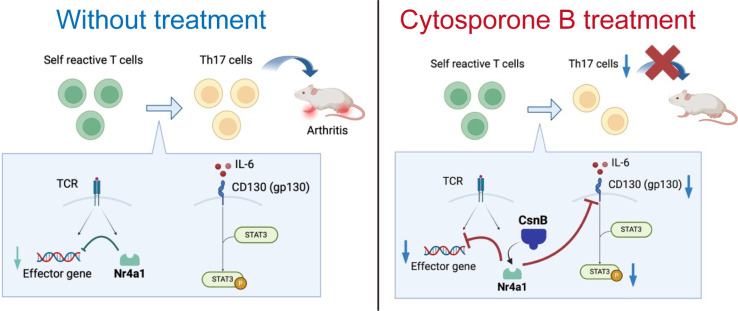

## 1 Introduction

Persistent synovial inflammation and progressive joint destruction are characteristic manifestations of rheumatoid arthritis (RA), a chronic inflammatory disease, typically driven by the interplay of various immune cells, including T cells, B cells, and monocytes (1). Among these, the notable association between RA and the human leukocyte antigen-DRB1 locus has been reported, underscoring the pivotal role of CD4^+^ T cells in the pathophysiology of the disease (2–4). Notably, CD4^+^ T helper (Th) cells have been classified into different subsets based on their functions, cytokine production, and chemokine receptor expression. Although the precise contributions of each CD4^+^ T cell subset to RA remain elusive, a pathogenic role of interleukin(IL)-17-producing Th17 cells has been reported in the development and progression of RA (5–7).

*Nr4a1*, a member of the nuclear receptor subfamily 4A (NR4A) family, is the most abundant among the three NR4A members in T cells (8). Acute stimulation of the T cell antigen receptor (TCR) leads to rapid upregulation of *Nr4a1*, peaking at 2–6 hours (9–11). Studies have reported that *Nr4a1* functions as a negative regulator of T-cell activation, as *Nr4a1*-deficient T cells exhibit enhanced effector functions and increased cytokine production (12). In addition to its role in peripheral T cell responses, NR4A has also been implicated in central tolerance owing to apoptosis induction in immature thymocytes during thymic negative selection (13–16). Autoreactive T cells are generally derived from those that escape thymic negative selection, suggesting a critical role of *Nr4a1* in preventing autoimmunity by controlling both central and peripheral T cell tolerance (17).

Additionally, *Nr4a1* is a known orphan receptor, and its functions have been reported in both ligand-dependent and ligand-independent manners (18). Although endogenous ligands have not been conclusively identified, several exogenous ligands have been reported (19–21). Cytosporone B (CsnB), an octaketide fungal metabolite, is a small-molecule agonist that directly binds to the ligand-binding domain of *Nr4a1*-encoded NUR77. Various studies have used CsnB to investigate the role of *Nr4a1* in various murine models of inflammatory diseases (22–25). For instance, in a dextran sulfate sodium-induced colitis model, CsnB was shown to ameliorate disease severity by modulating Toll-like receptor and IL-1 receptor signaling (25). Moreover, CsnB-mediated *Nr4a1* agonization has shown therapeutic efficacy in the experimental autoimmune encephalomyelitis model by suppressing the production of interferon (IFN)-γ and IL-17 in the central nervous system (24).

The SKG mouse strain can spontaneously develop chronic arthritis via an autoimmune mechanism, serving as a valuable model for studying human inflammatory arthritis (26). Histopathologically, SKG arthritis has been characterized by symmetrical, pannus-forming synovitis in limb joints, which resembles the joint pathology of RA (26). Although SKG mice can spontaneously develop arthritis under conventional housing environments, they require innate immune stimuli in specific pathogen-free conditions for disease pathogenesis. Reportedly, SKG mice harbor a hypomorphic mutation in *Zap70*, resulting in attenuated TCR signaling, which impairs thymic negative selection, thereby allowing the escape of autoreactive T cells into the periphery and exacerbating autoimmune pathology. In NUR77-enhanced green fluorescent protein SKG mouse model, the peripheral naïve CD4^+^ T cells have been shown to exhibit a higher NUR77 expression than that in wild-type mice (27). These studies suggest that SKG mice possess a subset of CD4^+^ T cells that are chronically stimulated by antigens, including autoantigens.

Many studies have suggested targeting *Nr4a1* as a potential approach to mitigate autoimmune arthritis in murine models, such as collagen-induced arthritis (CIA) and K/BxN serum transfer-induced arthritis (STIA) (23, 28, 29). In CIA mouse arthritis model, mice are immunized with type II collagen, which leads to the activation of CD4^+^ T cells and B cells, thereby initiating arthritis development (30). In contrast, in the STIA model, arthritogenic serum from K/BxN mice is transferred, and the pathogenesis is predominantly mediated by mechanisms independent of adaptive immunity (31). Although studies on these models have expanded on the role of *Nr4a1* agonization in inflammatory arthritis, its effects in T cell-dominant autoimmune arthritis remain elusive. Hence, this study aimed to investigate the therapeutic effect of CsnB-mediated *Nr4a1* agonization on arthritis development in SKG mice and evaluate the effect on T cell function.

## 2 Methods

### 2.1 Experimental animals

SKG mice were obtained from CLEA Japan, Inc. (Osaka, Japan) and bred in specific pathogen-free conditions under a climate-controlled facility with a 12-h light/dark cycle. All animal experiments were approved and performed in accordance with the guidelines of the Institutional Animal Care Committee at Kyoto University (approval numbers MedKyo25238, MedKyo16106–23104).

### 2.2 Induction, scoring, and treatment of arthritis

Female SKG mice (10–14-week-old) were intraperitoneally injected with 2 mg zymosan A (ZyA) (Sigma-Aldrich, Japan) to induce arthritis. Clinical arthritis scores were assessed as previously described (26) and were defined as follows: 0, absence of swelling or erythema; 0.1, presence of swelling or erythema in the digits; 0.5, mild swelling and/or erythema in the wrists or ankle joints; and 1, severe swelling in larger joints. The total score for each mouse was obtained by summing the scores from all affected joints. SKG mice received intraperitoneal injections of 15 mg/kg CsnB (Sigma-Aldrich, Japan) or dimethyl sulfoxide (DMSO) thrice per week, beginning 1 d after ZyA administration.

### 2.3 Flow cytometry

For flow cytometry, single-cell suspensions of splenocytes were harvested from the spleens of SKG mice. Additionally, the synovial tissues from the joints were dissected into small fragments and digested enzymatically for 30 min at 37 °C in Roswell Park Memorial Institute-1640 medium, containing collagenase type I and IV (Worthington Biochemical, US). Following incubation, the digested tissues were mechanically dissociated and filtered through a 70-μm mesh strainer to obtain single-cell suspensions of synovial cells. The monoclonal antibodies used for flow cytometry are presented in **Supplementary Table S1**. For intracellular staining of transcription factors, the FoxP3 staining buffer set (eBioscience) was used according to the manufacturer’s instructions. For intracellular staining of cytokines, cells were stimulated in Roswell Park Memorial Institute-1640 medium supplemented with 10% serum, 1% ×100 non-essential amino acids, 10 mM N-2-hydroxyethylpiperazine-N′-2-ethanesulfonic acid buffer, 1 mM sodium pyruvate, 2 mM L-glutamine, and 50 μM b-mercaptoethanol for 4 h with 20 ng/mL phorbol 12-myristate 13-acetate (Sigma-Aldrich) and 1 mM ionomycin (Sigma-Aldrich) in the presence of GolgiStop^™^ (BD Bioscience). After stimulation, cells were fixed and permeabilized using BD Cytofix/Cytoperm^™^ (BD Bioscience) according to the manufacturer’s instructions. Flow cytometric data were acquired and analyzed using an LSRFortessa^™^ cell analyzer (BD Biosciences) and the FlowJo software (TreeStar).

### 2.4 *In vitro* T cell stimulation with anti-CD3/anti-CD28 antibodies

For *in vitro* stimulation, CD4^+^ T cells were purified using a magnetic-activated cell sorting system with the CD4^+^ T Cell Isolation Kit (Miltenyi Biotec) according to the manufacturer’s instructions. Flat-bottom 96-well plates were coated with 1 μg/mL anti-CD3 antibody (clone 2c11, BioLegend) and 2 μg/mL anti-CD28 antibody (clone 37.51, BioLegend) at 4 °C overnight. Following incubation, cells were plated (5 × 10^5^ cells/well) in the culture media containing 10 ng/mL CsnB or DMSO for 2 or 16 h.

### 2.5 RNA sequencing

CD4^+^ T cells were cultured on anti-CD3/anti-CD28-coated plates for 2 h; following this, the cells were lysed in RLT buffer supplemented with 1% β-mercaptoethanol. Next, the total RNA was extracted using the RNeasy Plus Kit (QIAGEN) according to the manufacturer’s instructions. Messenger RNA (mRNA) libraries were prepared using the Illumina TruSeq Stranded mRNA Library Preparation Kit and sequenced on the NovaSeq X Plus platform by Marogen Japan Co., Ltd. (Tokyo, Japan). Trimmed FASTQ files were aligned to the reference genome using HISAT2, and transcript assembly was performed using StringTie. RNA sequencing was performed using biological triplicates for each experimental condition. Differential gene expression between DMSO- and CsnB-treated CD4^+^ T cells was analyzed using the DeSeq2 on R ver. 4.4.2. Gene set enrichment analysis (GSEA) was performed using ranked lists of all expressed genes derived from DESeq2 analysis. Genes were ranked according to log2 fold change, and enrichment analysis was conducted using the clusterProfiler package with Gene Ontology biological process gene sets. Gene sets with an adjusted P value < 0.05 were considered significantly enriched.

### 2.6 *In vitro* Th1/17 differentiation

For *in vitro* Th1/17 differentiation, naïve CD4^+^ T cells were isolated using the magnetic-activated cell sorting system and the naïve CD4^+^ T Cell Isolation Kit (Miltenyi Biotec) according to the manufacturer’s instructions. Purified cells were cultured on anti-CD3/anti-CD28 antibody-coated plates in the presence of 20 ng/mL IL-6, IL-23, and IL-1β (R&D Systems) to induce Th17 differentiation (32). The differentiation medium was supplemented with 10 ng/mL CsnB or DMSO. After 4 days of incubation, flow cytometry was performed to assess the harvested cells for cytokine production.

### 2.7 Statistical analyses

Statistical analyses and data visualizations were performed using Prism v10 (GraphPad Software, Inc.). Data are presented as mean ± standard error of the mean, unless otherwise specified. Unpaired or paired Student’s t-test was used to assess the significance of differences between the two groups, and corrections for multiple comparisons across time points or doses were applied using the Holm–Šídák method. Significance was defined as * P < 0.05, and **P < 0.01.

## 3 Results

### 3.1 CsnB mitigated arthritis development in SKG mice

**Figure 1:**
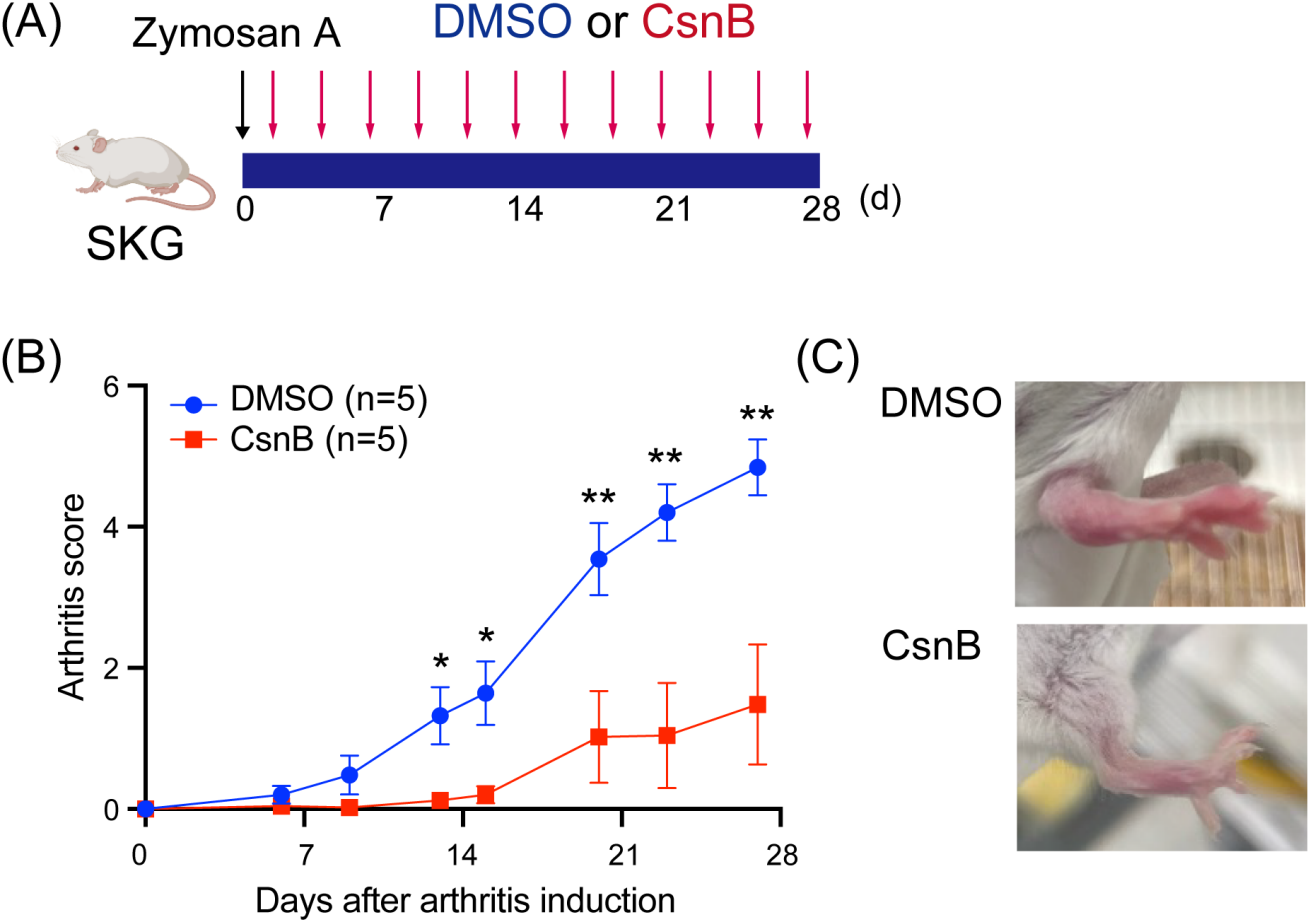
Cytosporone B (CsnB) inhibits arthritis development in SKG mice. **(A)** CsnB (15 mg/kg, three times weekly) was administered starting 1 day after arthritis induction with a single intraperitoneal injection of 2 mg zymosan **(A, B)** Clinical scores were significantly reduced in CsnB-treated mice compared with dimethyl sulfoxide (DMSO) controls. Data are representative of three independent experiments with n = 5 mice per group. **(C)** Representative image of swollen paws from CsnB- and DMSO-treated mice. *P < 0.05, **P < 0.01.

The therapeutic effect of CsnB on the experimental arthritis model was evaluated in SKG mice, which develop autoimmune arthritis following innate immune stimulation with ZyA, curdlan, or mannan (26). Following 1 day after the intraperitoneal injection of ZyA (200 μg per mouse), 15 mg/kg CsnB was administered intraperitoneally thrice per week (n = 5 each) (**Figure 1A**). CsnB treatment delayed the onset of arthritis and significantly reduced its severity by day 28 compared with that in the control group (**Figures 1B, C**). These findings suggest that intraperitoneal treatment with CsnB attenuates the development of experimental arthritis *in vivo*.

### 3.2 Effector memory T cells and Th17 cells were decreased in CsnB-treated mice

**Figure 2:**
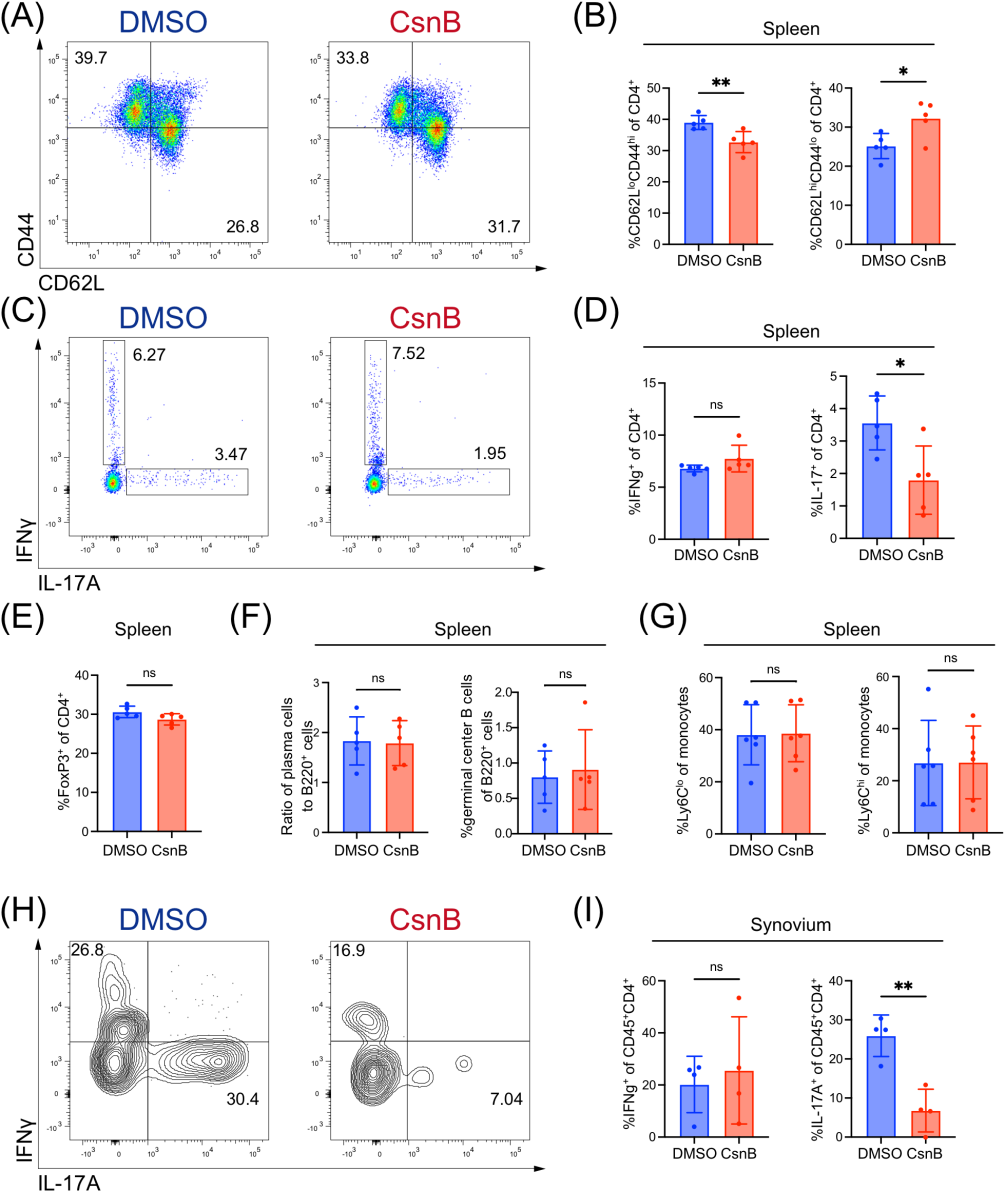
Reduction of T helper (Th)17 cells in spleen and synovium after cytosporone B (CsnB) treatment. **(A)** Representative flow plots showing naïve (CD4^+^CD62LhiCD44lo) and effector memory (CD4^+^CD62LloCD44hi) T cells in the spleen. **(B)** Frequency of naïve and effector memory T cells among CD4^+^ T cells. **(C)** Flow plots showing Th1 (CD4^+^IFN-γ^+^) and Th17 (CD4^+^IL-17^+^) cells in the spleen. **(D)** Frequency of Th1 and Th17 cells among splenic CD4^+^ T cells. **(E)** Frequency of T regulatory (CD4^+^CD25^+^FoxP3^+^) cells among CD4^+^ T cells. **(F)** Ratio of plasma cells (CD138^+^) to total B cells (left) and frequency of germinal center B cells (B220^+^IgDloGL-7^+^Fas^+^) among total B cells (right). **(G)** Frequency of Ly6Clo and Ly6Chi monocytes. **(H)** Flow plots of Th1 and Th17 cells in synovium. **(I)** Frequency of Th1 and Th17 cells among synovial CD4^+^ T cells. Graphs show mean ± standard error of the mean. Unpaired t-test; *P < 0.05, **P < 0.01. ns means not significant.

The immunological mechanisms underlying the alleviation of arthritis in CsnB-treated mice were investigated. SKG mice harbor a point mutation in *Zap70*, which leads to an increase in the number of autoreactive T cells in the periphery. Therefore, flow cytometric analysis of splenic T cell subsets isolated from CsnB-treated mice was performed (26). The total number of splenic CD4^+^ T cell was not significantly different between the groups, indicating that CsnB did not induce lymphopenia (**Supplementary Figure S1A**). Notably, the proportion of effector memory CD4^+^ T cells (CD4^+^CD62L^–^CD44^+^) reduced in the CsnB-treated group, whereas that of naïve CD4^+^ T cells (CD4^+^CD62L^+^CD44^–^) increased (**Figures 2A, B**). Furthermore, the frequency of IFNγ^+^CD4^+^ T cells remained unchanged, whereas that of IL-17A^+^CD4^+^ T cells decreased in the spleen of CsnB-treated mice (**Figures 2C, D**). When analyzed as absolute counts per spleen, the differences in naïve and effector-memory CD4^+^ T cells were diminished and did not reach statistical significance (**Supplementary Figure S1B**), indicating that the observed changes reflect redistribution within the CD4^+^ compartment rather than true expansion or contraction of these subsets. In contrast, IL-17A^+^ CD4^+^ T cells were reduced in both frequency and absolute number, whereas IFNγ^+^ CD4^+^ T cell numbers remained unchanged (**Supplementary Figure S1C**). Additionally, the results showed that the frequencies of regulatory T cells, germinal center B cells, plasma cells, and monocytes did not exhibit significant differences between the CsnB-treated and control groups (**Figures 2E–G**). In the synovium, the proportion of IL-17A^+^ CD4^+^ T cells reduced in the CsnB-treated mice, whereas the proportion of IFNγ^+^ CD4^+^ T cells remained unaltered (**Figures 2H, I**). Conversely, absolute numbers of both IL-17A^+^ and IFNγ^+^ CD4^+^ T cells were diminished in CsnB-treated mice, suggesting that total effector T cell accumulation within the joint was inhibited, with a more pronounced attenuation of Th17 polarization (**Supplementary Figure S1D**). Overall, these findings suggest that CsnB treatment modulates the population of peripheral CD4^+^ T cells, particularly by suppressing pro-inflammatory Th17 responses.

### 3.3 CsnB inhibits T cell activation following anti-CD3 and anti-CD28 antibody stimulation

**Figure 3:**
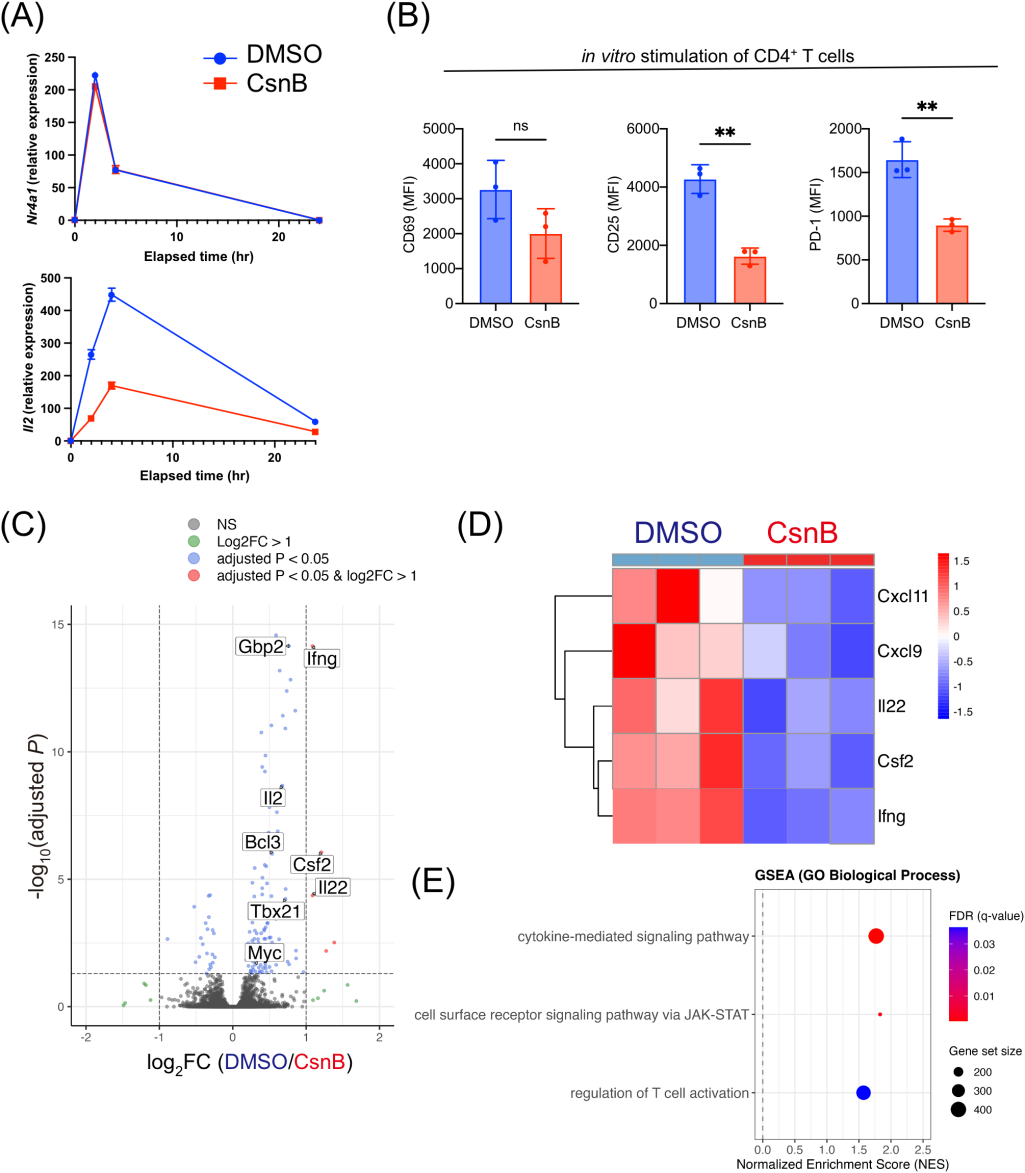
Cytosporone B (CsnB) attenuates T cell activation induced by anti-CD3/CD28 stimulation. **(A)** Time-course of *Nr4a1* and *Il2* expression in CD4^+^ T cells after anti-CD3/CD28 stimulation. **(B)** Quantification of CD69, CD25, and PD-1 mean fluorescence intensity in CD4^+^ T cells after 16 h on anti-CD3/CD28-coated plates. **(C)** Volcano plot of RNA sequencing results 2 h after stimulation, comparing dimethyl sulfoxide (DMSO) and CsnB conditions (Data were generated from biological triplicates). Genes associated with T cell activation and adjusted P value < 0.05 are shown. **(D)** Heatmaps showing genes significantly downregulated in CsnB-treated samples (genes with adjusted P value < 0.05 and log2 fold change ≥ 1). **(E)** Representative immune-related biological processes identified by gene set enrichment analysis using ranked gene lists are shown. Dot size indicates gene set size, and color denotes FDR. **P < 0.01.

*In vitro* analysis was performed to elucidate the role of CsnB during TCR stimulation. CD4^+^ T cells were isolated from SKG mice and cultured on anti-CD3/anti-CD28 antibody-coated plates in the presence or absence of CsnB. Quantitative polymerase chain reaction analysis revealed that *Nr4a1* mRNA expression peaked at 2 h post-TCR stimulation, and *Il2* expression peaked at 4 h (**Figure 3A**). CsnB treatment substantially suppressed *Il2* expression during TCR stimulation and significantly inhibited the upregulation of activation markers post-TCR stimulation, including CD25 and PD-1 (**Figure 3B**). Based on the observation that *Nr4a1* expression peaked at 2 h after stimulation, RNA sequencing was performed at this early time point to capture CsnB-induced modulation of early TCR-driven transcriptional responses. For RNA sequencing, transcriptomic analysis revealed that CsnB downregulated the expression of inflammation-related genes, including *Ifng*, *Csf2*, and *Il22* (**Figures 3C, D**). GSEA revealed that pathways related to cytokine-mediated signaling, T cell activation, and JAK–STAT signaling were significantly de-enriched in CsnB-treated samples compared with controls (**Figure 3E**). Other differentially expressed genes and GSEA results are described in **Supplementary Tables 2, 3**. Overall, these findings suggest that CsnB attenuates T cell activation in response to TCR signaling.

### 3.4 CsnB inhibits Th17 differentiation and IL-6 signals

**Figure 4:**
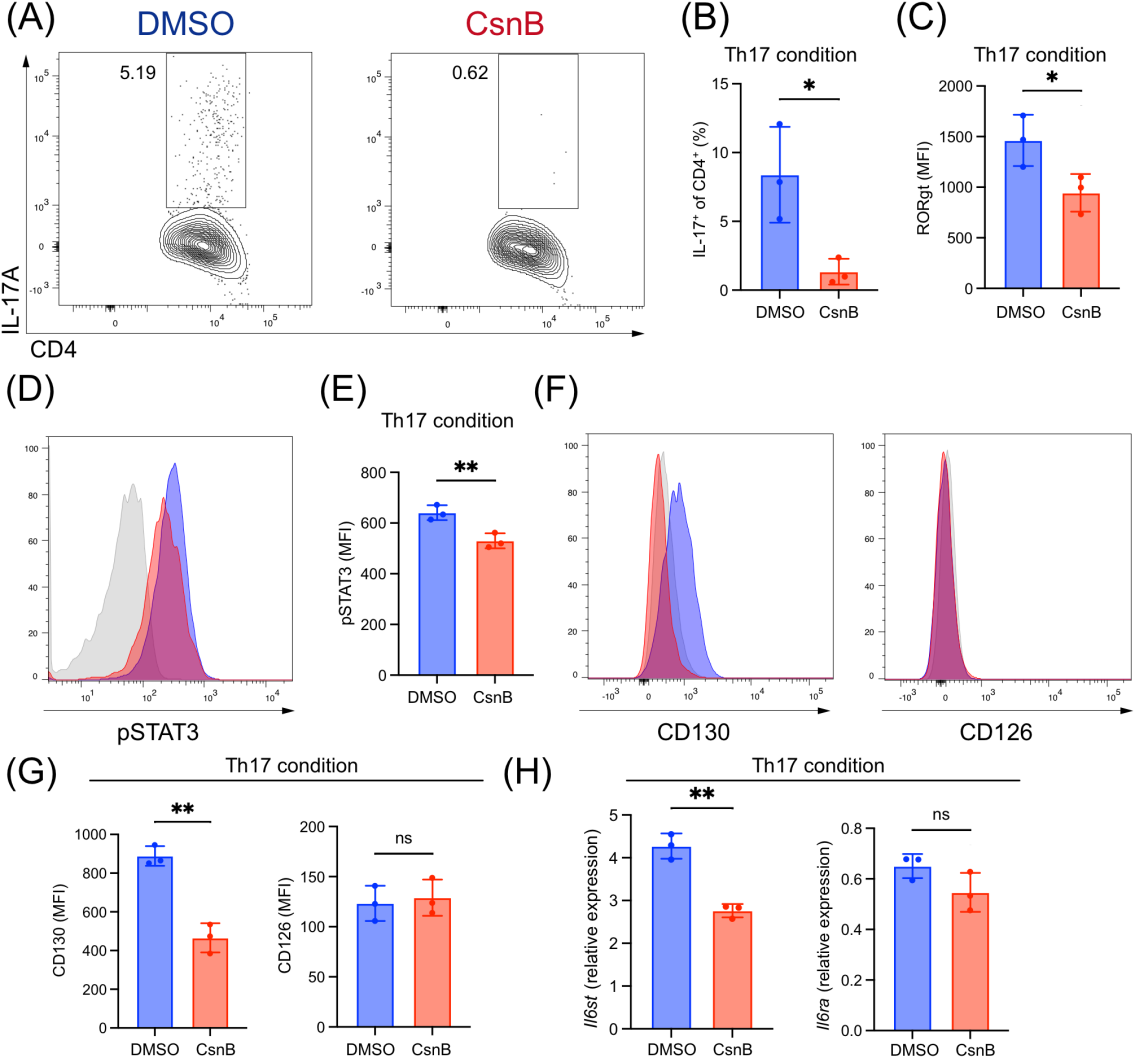
Cytosporone B (CsnB) inhibits T helper (Th)17 differentiation and signal transducer and activator of transcription 3 (STAT3)/CD130 expression *in vitro*. **(A)** Representative plots of Th17 cells after 4 d of naïve CD4^+^ T cell culture under Th17-polarizing conditions (interleukin [IL]-6 + IL-1β + IL-23). **(B)** Frequency of Th17 cells among CD4^+^ T cells. **(C)** Retinoic acid-related orphan receptor-γt expression quantification after 4 d of Th17 culture. **(D)** Flow plots of phosphorylated STAT3 in CD4^+^ T cells after 2 d **(E)** Quantification of phosphorylated STAT3 after 2 d **(F)** Flow plots of CD130 and CD126 expression after 2 d **(G)** Quantification of CD130 and CD126 after Th17 culture. **(H)***Il6st* and *Il6ra* expression in CD4^+^ T cells after 2 h of culture. *P < 0.05, **P < 0.01.

We hypothesized that CsnB inhibits Th17 differentiation *in vivo*. Accordingly, the effect of CsnB was evaluated on Th17 differentiation *in vitro*, and naïve CD4^+^ T cells isolated from SKG mice were cultured under Th17-polarizing conditions (IL-6, IL-1β, and IL-23) in the presence or absence of CsnB. Strikingly, CsnB treatment significantly reduced the proportion of IL-17A^+^CD4^+^ cells (**Figures 4A, B**) and retinoic acid-related orphan receptor (ROR)γt expression, a key transcription factor for Th17 differentiation, as assessed by flow cytometry (**Figure 4C**). Th17 differentiation was not inhibited in naïve CD4^+^ T cells isolated from *Nr4a1*-knockout mice (**Supplementary Figure S2**). IL-6 plays a critical role in Th17 cell differentiation; therefore, the effect of CsnB on IL-6 signal transduction was investigated (33). Phospho-flow cytometry results of signal transducer and activator of transcription 3 (STAT3) phosphorylation revealed that CsnB treatment significantly reduced STAT3 phosphorylation during Th17 differentiation (**Figures 4D, E**). The analysis of IL-6 receptor component expression, including the ligand-binding IL-6Rα-chain (CD126) and signal-transducing subunit gp130 (CD130), showed that CsnB significantly reduced CD130 expression; CsnB did not affect CD126 expression (**Figures 4F, G**). Furthermore, CsnB treatment reduced relative mRNA expression of *Il6st* (CD130), whereas *Il6ra* (CD126) expression remained unchanged (**Figure 4H**). Overall, these results suggest that CsnB inhibits CD130 transcription and reduces IL-6 signal transduction during Th17 differentiation.

## 4 Discussion

Herein, the therapeutic potential of targeting *Nr4a1* with its specific agonist, CsnB, was investigated in a mouse model of autoimmune arthritis. Of note, CsnB treatment significantly attenuated arthritis development in SKG mice (**Supplementary Figure S3**). Moreover, the frequency of Th17 cells was reduced in both the spleen and synovium of CsnB-treated mice, whereas that of other immune cell populations remained unaffected. *In vitro* analyses revealed that CsnB modulated T cell activation in response to stimulation with anti-CD3 and anti-CD28 antibodies. Furthermore, CsnB could inhibit Th17 differentiation by blocking the IL-6 signaling pathway.

Targeting *Nr4a1* has been reported to ameliorate autoimmune arthritis in both CIA and STIA models (23, 28, 29). However, these models represent immunization/antibody-induced acute forms of arthritis and are self-limiting. In contrast, SKG mice can develop chronic autoimmune arthritis following innate immune stimulation under the specific pathogen-free environment, without spontaneous resolution (26). To the best of our knowledge, the effects of *Nr4a1* agonization on T cells in this chronic model of autoimmune arthritis have not been previously characterized. In the present study, CsnB treatment reduced the population of effector memory T cells, with specific inhibition of Th17 cells, as revealed by the subset analysis; Th1 cell population remained unaffected. This finding is consistent with that of previous studies, which showed that *Nr4a1* overexpression in T cells did not affect Th1 cytokines in the CIA model; however, the impact on Th17 cytokines was not evaluated in previous studies (28). Considering the predominant role of Th17 cells in arthritis pathogenesis in SKG mice (34), the results of the present study suggest that inhibiting Th17 differentiation is a key mechanism underlying the therapeutic effect of CsnB in this model.

A study on *Nr4a1*-deficient mice demonstrated that *Nr4a1* functions as a negative regulator of T-cell activation (12). Upregulated expression of activation markers such as CD25 and CD69 was observed in the reported mice, along with a high population of effector memory T cells in the absence of exogenous stimulation. Conversely, *Nr4a1* overexpression in CD4^+^ T cells resulted in reduced transcription of *Il2, Ifng*, and *Tbx21* (35). *Nr4a1* expression was transiently upregulated within a few hours following TCR stimulation, albeit it returned to the baseline within 24 hours (36). In the present study, CsnB inhibited the expression of T cell activation markers during the acute phase of TCR signaling, thereby suggesting that CsnB enhances *Nr4a1* activity and reinforces its role in negative regulation of T cell activation.

In this study, CsnB significantly inhibited the *in vitro* differentiation of naïve CD4^+^ T cells into Th17 cells, which is consistent with findings from a previous study (24). Furthermore, CsnB treatment attenuated STAT3 phosphorylation during Th17 differentiation. STAT3 is an essential transcription factor implicated in Th17 differentiation owing to its role in the expression of various downstream genes, including *Rorc*, which encodes RORγt, the master regulator of the Th17 lineage (37). STAT3 phosphorylation is primarily mediated by IL-6 signaling through its receptor components, such as IL-6α and the common subunit gp130. CsnB could suppress gp130 expression. The IL-6-gp130-Janus kinase signaling axis is the principal pathway for STAT3 activation; hence, gp130 downregulation likely contributed to the STAT3 phosphorylation and subsequent decrease in RORγt expression. A recent study reported that gp130 expression is induced by TCR signaling (38), which suggests that CsnB may act on a downstream component of the TCR pathway to repress gp130 expression. In our analyses, although CsnB treatment mitigated *Il6st* expression, it remains unclear whether this regulation is direct or indirect. The observed reduction in *Il6st* expression may occur through indirect mechanisms, including transcriptional repression via *Nr4a1*-associated regulatory networks, modulation of upstream cytokine signaling, or epigenetic remodeling associated with altered T cell activation states. Collectively, these findings indicate the interference of CsnB with Th17 differentiation via the modulation of both TCR and IL-6 signaling cascades.

Although the findings of this study suggest that CsnB represses Th17 differentiation through *Nr4a1* agonization, there are some discrepancies with a previous study reporting that Th17 cell differentiation remained unaffected in *Nr4a* triple-knockout (*Nr4a1/2/*3) mice (39). Additionally, enhanced Th17 differentiation has been shown in *Nr4a1* single-knockout mice in the experimental autoimmune encephalomyelitis model (40). *Nr4a2* knockdown by small interfering RNA has been reported to suppress Th17 differentiation *in vitro* (41). This functional redundancy among *Nr4a* family members suggests that the suppressive effect of CsnB on Th17 differentiation may be the result of a cumulative modulation of multiple *Nr4a* factors and related transcriptional networks. CsnB was originally identified as a naturally occurring agonist of *Nr4a1* that directly binds to its ligand-binding domain and activates *Nr4a1*-dependent transcriptional activity (19). However, although some studies showed the effect of CsnB was impaired in *Nr4a1*-knockout mice, its functional selectivity toward *Nr4a1* versus other *Nr4a* family members at the doses used *in vivo* and *in vitro* experiments in our study and previous studies has not been fully characterized, and potential contributions of *Nr4a2* or *Nr4a3* cannot be excluded (24, 42). SKG mice harbor a hypomorphic mutation in *Zap70*, which encodes a cytoplasmic tyrosine kinase essential for initiating proximal TCR signal transduction (26). Consequently, impaired TCR signaling can lead to the escape of autoreactive T cells from negative selection in the thymus, allowing them to persist in the periphery. Upon being chronically exposed to self-antigens, these autoreactive T cells exhibit upregulated *Nr4a1* expression in CD4^+^ T cells (27). IL-6 signaling was enhanced in the *Nr4a1*^hi^ CD4^+^ T cells, and Th17 cells were more prevalent in adoptive transfer models using *Nr4a1*^hi^ CD4^+^ T cells. CsnB is a naturally occurring *Nr4a1* agonist and is presumed to exert greater effects on *Nr4a1*^hi^ CD4^+^ T cells, thereby modulating their pathogenic potential.

Potential risks of systemic *Nr4a1* activation should be considered. *Nr4a* family members have been implicated as key regulators of T cell tolerance and exhaustion, restraining effector cytokine programs under chronic antigen stimulation (35). Consistent with this concept, pharmacological activation of *Nr4a1* signaling could, in principle, increase susceptibility to infections, dampen vaccine responses, or compromise tumor immune surveillance (35, 43). Importantly, however, the immunological consequences of *Nr4a1* agonization appear to be context-dependent. For example, administration of the CsnB improved outcomes in an influenza infection model, reducing lung viral loads and improving pulmonary function (42). Therefore, future studies should define dose- and time-dependent safety profiles of *Nr4a1* agonization and evaluate immune competence longitudinally to balance therapeutic benefit with systemic immune risks.

It has been reported that approximately 20% of patients with RA possess antibodies and self-reactive T cells that act against one of the self-antigens identified in SKG mice (44). Moreover, a subset of patients with RA has presented with abnormal TCR signaling, similar to that observed in SKG mice, particularly regarding defective central tolerance and the persistence of autoreactive T cells. Genetic studies implicate altered proximal TCR signaling pathways in RA susceptibility, as indicated by the *PTPN22* risk variant, which is linked to reduced TCR signaling (45, 46). In addition, some populations show synovium dominated by lymphoid lineage infiltration, including T cells (47). CsnB could interfere with TCR signaling and suppress effector T cell activation. This mechanism is especially relevant in RA, where both effector memory T cells and Th17 cells contribute to the chronicity of the disease. The findings of this study, showing that CsnB ameliorates arthritis and reduces effector T cell subsets in SKG mice, highlight its potential as a novel immunomodulatory agent for treating autoimmune arthritis, particularly in patients with TCR-signaling-defective or T-cell-dominant mechanisms.

This study has some limitations. First, although the therapeutic efficacy of CsnB in the SKG mouse model of autoimmune arthritis was investigated, the generalizability of these findings to other animal models or humans remains uncertain. Arthritis in SKG mice is induced by innate immune stimulation and is characterized by TCR signaling defects due to the hypomorphic *Zap70* mutation, which may not fully recapitulate the diverse immunopathogenic mechanisms of RA across diverse patient subpopulations. Second, although clinical scoring reliably reflects inflammatory disease activity in the SKG model, this study lacked histological analyses. The present findings focus on immunological modulation of arthritis rather than definitive structural joint protection, and future studies incorporating joint histopathology will be necessary to further support these effects. Third, although the results show that CsnB suppressed IL-6 signaling by downregulating CD130 (*Il6st*), the upstream molecular mechanisms by which *Nr4a1* modulates CD130 transcription remain unclear. Hence, further studies involving transcriptional and epigenetic profiling are warranted to delineate the regulatory network involved with CsnB and arthritis.

In conclusion, the findings of this study demonstrate that CsnB, one of the agonists of *Nr4a1*, can suppress T cell activation and Th17 differentiation, thereby leading to the attenuation of autoimmune arthritis in SKG mice. Altogether, these findings highlight *Nr4a1* as a promising therapeutic target for T cell-mediated autoimmune arthritis.

## Data Availability

The datasets presented in this study can be found in online repositories. The names of the repository/repositories and accession number(s) can be found below: https://www.ncbi.nlm.nih.gov/geo/, GSE312091.

## References

[1] Smolen JS Aletaha D McInnes IB . Rheumatoid arthritis. Lancet. (2016) 388:2023–38. doi: 10.1016/S0140-6736(16)30173-8, PMID: 27156434

[2] Plenge RM Seielstad M Padyukov L Lee AT Remmers EF Ding B . TRAF1-C5 as a risk locus for rheumatoid arthritis – A genomewide study. N Engl J Med. (2007) 357:1199–209. doi: 10.1056/NEJMoa073491, PMID: 17804836 PMC2636867

[3] Hughes LB Reynolds RJ Brown EE Kelley JM Thomson B Conn DL . Most common single-nucleotide polymorphisms associated with rheumatoid arthritis in persons of European ancestry confer risk of rheumatoid arthritis in African Americans. Arthritis Rheum. (2010) 62:3547–53. doi: 10.1002/art.27732, PMID: 21120996 PMC3030622

[4] Mellado M Martínez-Muñoz L Cascio G Lucas P Pablos JL Rodríguez-Frade JM . T cell migration in rheumatoid arthritis. Front Immunol. (2015) 6:384. doi: 10.3389/fimmu.2015.00384, PMID: 26284069 PMC4515597

[5] Chabaud M Durand JM Buchs N Fossiez F Page G Frappart L . Human interleukin-17: A T cell-derived proinflammatory cytokine produced by the rheumatoid synovium. Arthritis Rheum. (1999) 42:963–70. doi: 10.1002/1529-0131(199905)42:5<963::AID-ANR15>3.0.CO;2-E, PMID: 10323452

[6] Leipe J Grunke M Dechant C Reindl C Kerzendorf U Schulze-Koops H . Role of Th17 cells in human autoimmune arthritis. Arthritis Rheum. (2010) 62:2876–85. doi: 10.1002/art.27622, PMID: 20583102

[7] Ziolkowska M Koc A Luszczykiewicz G Ksiezopolska-Pietrzak K Klimczak E Chwalinska-Sadowska H . High levels of IL-17 in rheumatoid arthritis patients: Il-15 triggers *in vitro* IL-17 production via cyclosporin A-sensitive mechanism. J Immunol. (2000) 164:2832–8. doi: 10.4049/jimmunol.164.5.2832, PMID: 10679127

[8] Hiwa R Brooks JF Mueller JL Nielsen HV Zikherman J . NR4A nuclear receptors in T and B lymphocytes: Gatekeepers of immune tolerance. Immunol Rev. (2022) 307:116–33. doi: 10.1111/imr.13072, PMID: 35174510

[9] Jennings E Elliot TAE Thawait N Kanabar S Yam-Puc JC Ono M . Nr4a1 and Nr4a3 reporter mice are differentially sensitive to T cell receptor signal strength and duration. Cell Rep. (2020) 33:108328. doi: 10.1016/j.celrep.2020.108328, PMID: 33147449 PMC7653457

[10] Moran AE Holzapfel KL Xing Y Cunningham NR Maltzman JS Punt J . T cell receptor signal strength in Treg and iNKT cell development demonstrated by a novel fluorescent reporter mouse. J Exp Med. (2011) 208:1279–89. doi: 10.1084/jem.20110308, PMID: 21606508 PMC3173240

[11] Tan C Hiwa R Mueller JL Vykunta V Hibiya K Noviski M . NR4A nuclear receptors restrain B cell responses to antigen when second signals are absent or limiting. Nat Immunol. (2020) 21:1267–79. doi: 10.1038/s41590-020-0765-7, PMID: 32868928 PMC8081071

[12] Liebmann M Hucke S Koch K Eschborn M Ghelman J Chasan AI . Nur77 serves as a molecular brake of the metabolic switch during T cell activation to restrict autoimmunity. Proc Natl Acad Sci U.S.A. (2018) 115:E8017–26. doi: 10.1073/pnas.1721049115, PMID: 30072431 PMC6112725

[13] Liu ZG Smith SW McLaughlin KA Schwartz LM Osborne BA . Apoptotic signals delivered through the T-cell receptor of a T-cell hybrid require the immediate-early gene nur77. Nature. (1994) 367:281–4. doi: 10.1038/367281a0, PMID: 8121494

[14] Calnan BJ Szychowski S Chan FK Cado D Winoto A . A role for the orphan steroid receptor Nur77 in apoptosis accompanying antigen-induced negative selection. Immunity. (1995) 3:273–82. doi: 10.1016/1074-7613(95)90113-2, PMID: 7552993

[15] Nielsen HV Yang L Mueller JL Ritter AJ Hiwa R Proekt I . Nr4a1 and Nr4a3 redundantly control clonal deletion and contribute to an anergy-like transcriptome in auto-reactive thymocytes to impose tolerance in mice. Nat Commun. (2025) 16:784. doi: 10.1038/s41467-025-55839-5, PMID: 39824797 PMC11742425

[16] Hiwa R Nielsen HV Mueller JL Mandla R Zikherman J . NR4A family members regulate T cell tolerance to preserve immune homeostasis and suppress autoimmunity. JCI Insight. (2021) 6:e151005. doi: 10.1172/jci.insight.151005, PMID: 34343134 PMC8492309

[17] Ohashi PS . Negative selection and autoimmunity. Curr Opin Immunol. (2003) 15:668–76. doi: 10.1016/j.coi.2003.09.013, PMID: 14630201

[18] Wang Z Benoit G Liu J Prasad S Aarnisalo P Liu X . Structure and function of Nurr1 identifies a class of ligand-independent nuclear receptors. Nature. (2003) 423:555–60. doi: 10.1038/nature01645, PMID: 12774125

[19] Zhan Y Du X Chen H Liu J Zhao B Huang D . Cytosporone B is an agonist for nuclear orphan receptor Nur77. Nat Chem Biol. (2008) 4:548–56. doi: 10.1038/nchembio.106, PMID: 18690216

[20] Hu M Luo Q Alitongbieke G Chong S Xu C Xie L . Celastrol-induced Nur77 interaction with TRAF2 alleviates inflammation by promoting mitochondrial ubiquitination and autophagy. Mol Cell. (2017) 66:141–153.e6. doi: 10.1016/j.molcel.2017.03.008, PMID: 28388439 PMC5761061

[21] Chintharlapalli S Burghardt R Papineni S Ramaiah S Yoon K Safe S . Activation of Nur77 by selected 1,1-Bis(3’-indolyl)-1-(p-substituted phenyl)methanes induces apoptosis through nuclear pathways. J Biol Chem. (2005) 280:24903–14. doi: 10.1074/jbc.M500107200, PMID: 15871945

[22] Jiang Y Zeng Y Huang X Qin Y Luo W Xiang S . Nur77 attenuates endothelin-1 expression via downregulation of NF-κB and p38 MAPK in A549 cells and in an ARDS rat model. Am J Physiol Lung Cell Mol Physiol. (2016) 311:L1023–35. doi: 10.1152/ajplung.00043.2016, PMID: 27765761 PMC5206403

[23] Brunet A LeBel M Egarnes B Paquet-Bouchard C Lessard A-J Brown JP . NR4A1-dependent Ly6Clow monocytes contribute to reducing joint inflammation in arthritic mice through Treg cells. Eur J Immunol. (2016) 46:2789–800. doi: 10.1002/eji.201646406, PMID: 27600773

[24] Yu H-Z Zhu B-Q Zhu L Li S Wang L-M . NR4A1 agonist cytosporone B attenuates neuroinflammation in a mouse model of multiple sclerosis. Neural Regener Res. (2022) 17:2765–70. doi: 10.4103/1673-5374.339492, PMID: 35662227 PMC9165396

[25] Wu H Li X-M Wang J-R Gan W-J Jiang F-Q Liu Y . NUR77 exerts a protective effect against inflammatory bowel disease by negatively regulating the TRAF6/TLR-IL-1R signalling axis. J Pathol. (2016) 238:457–69. doi: 10.1002/path.4670, PMID: 26564988

[26] Sakaguchi N Takahashi T Hata H Nomura T Tagami T Yamazaki S . Altered thymic T-cell selection due to a mutation of the ZAP-70 gene causes autoimmune arthritis in mice. Nature. (2003) 426:454–60. doi: 10.1038/nature02119, PMID: 14647385

[27] Ashouri JF Hsu L-Y Yu S Rychkov D Chen Y Cheng DA . Reporters of TCR signaling identify arthritogenic T cells in murine and human autoimmune arthritis. Proc Natl Acad Sci U.S.A. (2019) 116:18517–27. doi: 10.1073/pnas.1904271116, PMID: 31455730 PMC6744919

[28] De Silva S Han S Zhang X Huston DP Winoto A Zheng B . Reduction of the incidence and severity of collagen-induced arthritis by constitutive Nur77 expression in the T cell lineage. Arthritis Rheum. (2005) 52:333–8. doi: 10.1002/art.20736, PMID: 15641076

[29] Saini A Mahajan S Bhagyaraj E Kalra R Nanduri R Gupta R . An accord of nuclear receptor expression in CD4+ T cells in rheumatoid arthritis. Immunohorizons. (2019) 3:402–11. doi: 10.4049/immunohorizons.1900043, PMID: 31439624

[30] Luan J Hu Z Cheng J Zhang R Yang P Guo H . Applicability and implementation of the collagen-induced arthritis mouse model, including protocols (Review). Exp Ther Med. (2021) 22:939. doi: 10.3892/etm.2021.10371, PMID: 34335888 PMC8290431

[31] Christensen AD Haase C Cook AD Hamilton JA . K/BxN serum-transfer arthritis as a model for human inflammatory arthritis. Front Immunol. (2016) 7:213. doi: 10.3389/fimmu.2016.00213, PMID: 27313578 PMC4889615

[32] Ghoreschi K Laurence A Yang X-P Tato CM McGeachy MJ Konkel JE . Generation of pathogenic T(H)17 cells in the absence of TGF-β signalling. Nature. (2010) 467:967–71. doi: 10.1038/nature09447, PMID: 20962846 PMC3108066

[33] Bettelli E Carrier Y Gao W Korn T Strom TB Oukka M . Reciprocal developmental pathways for the generation of pathogenic effector TH17 and regulatory T cells. Nature. (2006) 441:235–8. doi: 10.1038/nature04753, PMID: 16648838

[34] Hirota K Hashimoto M Yoshitomi H Tanaka S Nomura T Yamaguchi T . T cell self-reactivity forms a cytokine milieu for spontaneous development of IL-17+ Th cells that cause autoimmune arthritis. J Exp Med. (2007) 204:41–7. doi: 10.1084/jem.20062259, PMID: 17227914 PMC2118414

[35] Liu X Wang Y Lu H Li J Yan X Xiao M . Genome-wide analysis identifies NR4A1 as a key mediator of T cell dysfunction. Nature. (2019) 567:525–9. doi: 10.1038/s41586-019-0979-8, PMID: 30814730 PMC6507425

[36] Cunningham NR Artim SC Fornadel CM Sellars MC Edmonson SG Scott G . Immature CD4+CD8+ thymocytes and mature T cells regulate Nur77 distinctly in response to TCR stimulation. J Immunol. (2006) 177:6660–6. doi: 10.4049/jimmunol.177.10.6660, PMID: 17082578

[37] Wan C-K Andraski AB Spolski R Li P Kazemian M Oh J . Opposing roles of STAT1 and STAT3 in IL-21 function in CD4^+^ T cells. Proc Natl Acad Sci U.S.A. (2015) 112:9394–9. doi: 10.1073/pnas.1511711112, PMID: 26170288 PMC4522759

[38] Qin Z Wang R Hou P Zhang Y Yuan Q Wang Y . TCR signaling induces STAT3 phosphorylation to promote TH17 cell differentiation. J Exp Med. (2024) 221:e20230683. doi: 10.1084/jem.20230683, PMID: 38324068 PMC10849914

[39] Sekiya T Kagawa S Masaki K Fukunaga K Yoshimura A Takaki S . Regulation of peripheral Th/Treg differentiation and suppression of airway inflammation by Nr4a transcription factors. iScience. (2021) 24:102166. doi: 10.1016/j.isci.2021.102166, PMID: 33665581 PMC7907427

[40] Wang L-M Zhang Y Li X Zhang M-L Zhu L Zhang G-X . Nr4a1 plays a crucial modulatory role in Th1/Th17 cell responses and CNS autoimmunity. Brain Behav Immun. (2018) 68:44–55. doi: 10.1016/j.bbi.2017.09.015, PMID: 28962999

[41] Raveney BJE Oki S Yamamura T . Nuclear receptor NR4A2 orchestrates Th17 cell-mediated autoimmune inflammation via IL-21 signalling. PloS One. (2013) 8:e56595. doi: 10.1371/journal.pone.0056595, PMID: 23437182 PMC3578929

[42] Egarnes B Blanchet M-R Gosselin J . Treatment with the NR4A1 agonist cytosporone B controls influenza virus infection and improves pulmonary function in infected mice. PloS One. (2017) 12:e0186639. doi: 10.1371/journal.pone.0186639, PMID: 29053748 PMC5650162

[43] Chen J López-Moyado IF Seo H Lio C-WJ Hempleman LJ Sekiya T . NR4A transcription factors limit CAR T cell function in solid tumours. Nature. (2019) 567:530–4. doi: 10.1038/s41586-019-0985-x, PMID: 30814732 PMC6546093

[44] Ito Y Hashimoto M Hirota K Ohkura N Morikawa H Nishikawa H . Detection of T cell responses to a ubiquitous cellular protein in autoimmune disease. Science. (2014) 346:363–8. doi: 10.1126/science.1259077, PMID: 25324392 PMC5554397

[45] Begovich AB Carlton VEH Honigberg LA Schrodi SJ Chokkalingam AP Alexander HC . A missense single-nucleotide polymorphism in a gene encoding a protein tyrosine phosphatase (PTPN22) is associated with rheumatoid arthritis. Am J Hum Genet. (2004) 75:330–7. doi: 10.1086/422827, PMID: 15208781 PMC1216068

[46] Lee AT Li W Liew A Bombardier C Weisman M Massarotti EM . The PTPN22 R620W polymorphism associates with RF positive rheumatoid arthritis in a dose-dependent manner but not with HLA-SE status. Genes Immun. (2005) 6:129–33. doi: 10.1038/sj.gene.6364159, PMID: 15674368

[47] Humby F Lewis M Ramamoorthi N Hackney JA Barnes MR Bombardieri M . Synovial cellular and molecular signatures stratify clinical response to csDMARD therapy and predict radiographic progression in early rheumatoid arthritis patients. Ann Rheum Dis. (2019) 78:761–72. doi: 10.1136/annrheumdis-2018-214539, PMID: 30878974 PMC6579551

