# Supplementary material for: Alleviation of experimental arthritis in SKG mice through Nr4a1 agonization
Source: Front Immunol. 2026 Feb 25;17:1758616. doi: 10.3389/fimmu.2026.1758616 (PMC12976022; doi:10.3389/fimmu.2026.1758616)

Supplementary Material

Supplementary Figure S1. Absolute numbers of lymphocyte subsets in spleen and synovium after CsnB treatment. (A) Total splenic CD4⁺ T-cell counts per mouse. (B) Absolute numbers of naïve ($CD4^{+}CD62L^{hi}CD44^{lo}$) and effector-memory ($CD4^{+}CD62L^{lo}CD44^{hi}$) CD4⁺ T cells in the spleen. (C) Absolute numbers of Th1 and Th17 cells per spleen. (D) Absolute numbers of synovial Th1 and Th17 CD4⁺ T cells per joint. Graphs show mean ± SEM. Unpaired t-test; P values indicated (* $P < 0.05$).

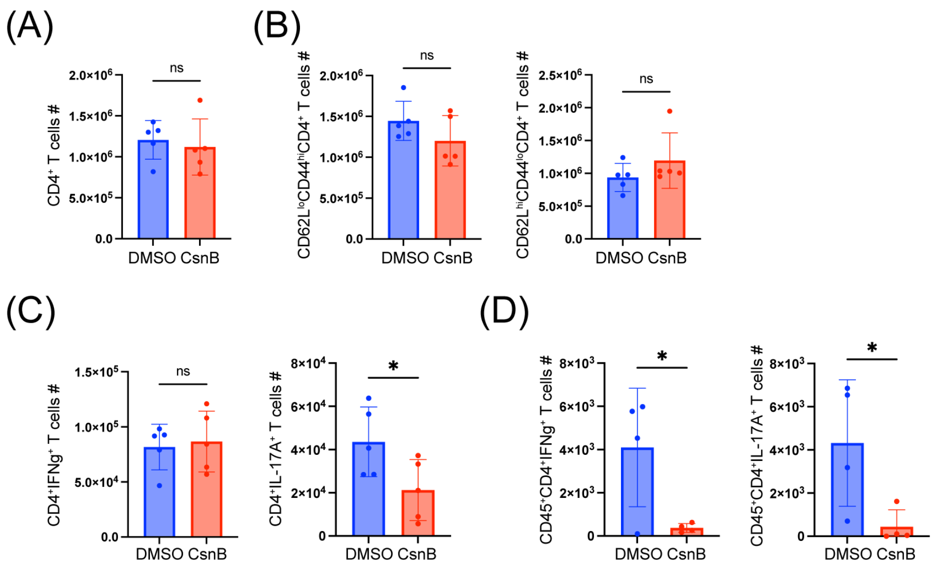

Supplementary Figure S2. Th17 differentiation was not inhibited by cytosporone B (CsnB) using naïve CD4$^+$ T cells derived from *Nr4a1*-knockout mice *in vitro*.

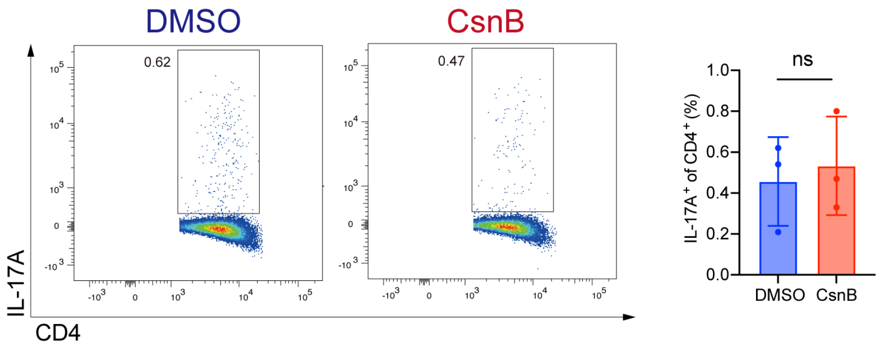

Supplementary Figure S3. Graphical abstract. Cytosporone B (CsnB) suppressed T cell receptor signalling and gp130 expression, thereby inhibiting Th17 differentiation. Arthritis development was also suppressed by CsnB.

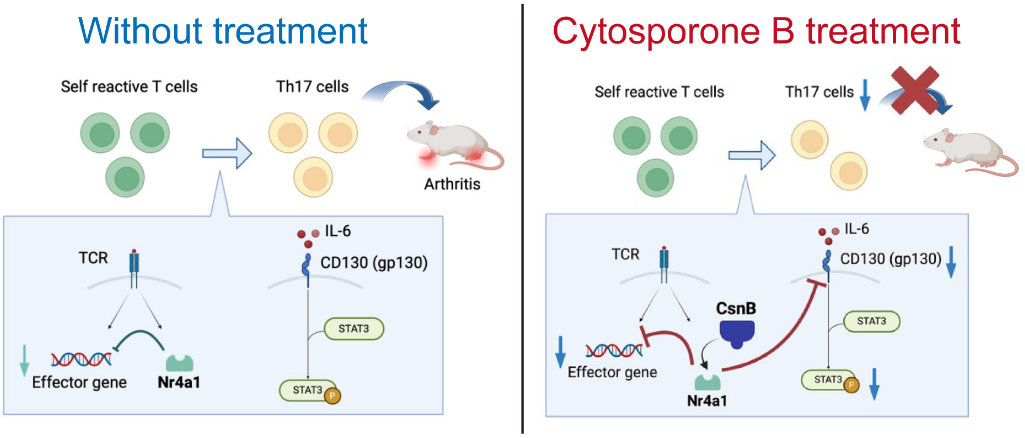

Supplement: Supplementary file 1 [file Image1.pdf]
